# A novel approach to the generation of seamless constructs for plant transformation

**DOI:** 10.1186/1746-4811-10-10

**Published:** 2014-05-10

**Authors:** Remy Kronbak, Christina Rønn Ingvardsen, Claus Krogh Madsen, Per Langkjær Gregersen

**Affiliations:** 1 Science and Technology, Department of Molecular Biology and Genetics, Aarhus University, Forsøgsvej 1, DK-4200 Slagelse, Denmark

**Keywords:** Plant transformation vector, linearization, Type IIS restriction endonucleases, Type IIB, In-Fusion™, Seamless cloning, Biolistics, Cereals, Transient expression

## Abstract

**Background:**

When creating plant transformation vectors, full control of nucleotides flanking the insert in the final construct may be desirable. Modern ligase-independent methods for DNA-recombination are based on linearization by classical type II restriction endonucleases (REs) alone or in combination with nicking enzymes leaving residual nucleotides behind in the final construct. We here explore the use of type IIS and type IIB REs for vector linearization that combined with sequence and ligase-independent cloning (SLIC) overcomes this problem and promotes seamless gene-insertion in vectors. Providing the basis for a collection of biolistic plant transformation vectors ready to be cloned with different genes-of-interest, we present two vectors, where promoter and terminator are joined by a spacer. During spacer-removal linearization (SRL), type IIS and type IIB REs remove their own recognition sequences from the vector leaving no undesired, short sequences behind.

**Results:**

We designed two plant transformation vectors prepared for SRL in combination with SLIC, pAUrumII and pAUrumIII, harboring a spacer with recognition sites for a type IIS and IIB RE, respectively. The gene for a green fluorescent protein, *gfp*, was successfully cloned into both vectors; traces of pAUrumIII, however, contaminated the transformation due to incomplete linearization, an issue not encountered with the type IIS linearized pAUrumII. Both constructs, pAUrumII-*gfp* and pAUrumIII-*gfp*, were functional, when tested *in vitro* on wheat and barley endosperm cells for transient *gfp* expression.

**Conclusions:**

All nucleotides flanking an insert in a biolistic plant transformation vector can be customized by means of SRL in combination with SLIC. Especially type IIS REs promote an efficient cloning result. Based on our findings, we believe that the SRL system can be useful in a series of plant transformation vectors, favoring the presence of functional sequences for optimal expression over redundant cloning-site remnants.

## Background

Combining DNA from various sources in single constructs typically for the purpose of over-expressing genes-of-interest is essential to modern research in fields like genetics, bioinformatics and biotechnology. Classical type II restriction endonucleases (REs) recognize and cleave short, palindromic sequences creating blunt or sticky ends (5’- or 3’-overhangs typically of one to a few nucleotides in length) and for decades they have been employed in a cleaving-and-ligating oriented manner. A range of limitations, however, accompanies this method. First, direction of the inserted fragment cannot be controlled unless the cloning is based on two REs creating different overhangs. Second, cloning of more than one fragment is not efficient and direction and order are not controllable using a single classical type II RE. Third, recognition sequences for the RE in question can only be present at the desired cloning location of the vector and flanking the insert, not elsewhere in the vector backbone or in the insert sequence. Fourth, ligase treatment of the inserted fragment and the backbone vector is required before transformation to *E. coli*. This is due to the fact that matching overhangs are most often not of more than four nucleotides in length. Such overhangs will likely dissociate at transformation temperatures, when non-ligated.

In recent years methods have been developed, which overcome most of the obstacles mentioned [1]. These methods are in general based on longer matching overhangs than most classical type II REs offer, and include ligase-independent cloning (LIC) [2], sequence and ligase-independent cloning (SLIC) [3] (equivalent to the commercially available In-Fusion™ cloning system, Clontech Laboratories, [4,5]), uracil specific excision reagent (USER™, New England Biolabs) cloning [6,7], circular polymerase extension cloning (CPEC) [8] and one-step isothermal *in vitro* recombination (Gibson Assembly™, Synthetic Genomics) [9]. The overhangs are typically at least 8–15 nt in size and remain sticky at transformation temperatures of 37–42°C independent of ligation. Several fragments can be assembled in the correct order and direction in a single reaction as long as all the sequences of homology involved (from which the matching overhangs are generated) are unique. In the In-Fusion™ system, which we use here, a poxvirus DNA polymerase displays 3’-5’ exonuclease activity against duplex DNA, when dNTP levels are low, due to its proofreading capacity. When the entire matching overhangs are created from 15 nt sequences of homology, spontaneous annealing of these overhangs occur, inhibiting further exonuclease activity of the polymerase. Thus, the assembled hybrid DNA only contains nicks or short gaps around the assembled sequences, which will be repaired and ligated by *E. coli* after transformation [4,5].

Meeting agricultural challenges, such as disease protection and drought tolerance, with the usage of genetically modified (GM) crops has not received great public acceptance because of the skepticism associated with the introduction of foreign DNA to an organism; an issue that intragenesis and cisgenesis have been developed to counteract (reviewed in [10,11]). For these two techniques, the gene pool, from which DNA can be introduced into an organism, is limited to crossable species and thus identical to the conventional breeder’s gene pool. Regardless of the GM technique employed, one might consider to avoid the presence of any unnecessary DNA sequence. Also in connection with gene fusion, there must be a seamless junction between a vector with an existing open reading frame (ORF) and an ORF-continuing insert if it is important to exclude additional amino acids between the fused proteins [12]. So for several reasons, it may be desired to fully control the nucleotides flanking the insert/inserts in the final construct, e.g. to have the gene-of-interest start codon at the exact location as the start codon of the native gene for the promoter in question, or to have only a Kozak consensus sequence in front of the gene for optimal mRNA translation or a tag sequence right after for tracking the expression of a gene, which has a high level of similarity to others. None of the mentioned cloning methods directly offer this, when the cloning is based on linearization by classical type II REs or a combination of REs and nicking enzymes (USER™ cassette) leaving residual nucleotides behind in the final construct. We explore here the use of type IIS and type IIB REs (reviewed in [13]) in the vector linearization process that, when combined with SLIC, overcomes this problem and creates seamless junctions. Type IIS REs are enzymes that recognize specific DNA sequences, but whereas classic type II REs cleave the DNA strands within their recognition sequence, type IIS cleave at a certain distance, most often a few base pairs away, from a non-palindromic recognition sequence. The type IIS RE *Eam1104I* has previously been used with LIC and SLIC for seamless gene fusion in *E. coli* expression vectors [14,15]. Dependent on the presence of S-adenosyl methionine (SAM), type IIB REs have the capability to cleave the DNA strands at both sides of the recognition sequence at defined distances. By using type IIS and type IIB REs for linearization, the recognition sequence can be separated from the cleaved sequence and in that way not reappear in the final construct.

Providing the basis for a collection of prepared plant transformation vectors, we here present two vectors, where promoter and terminator are joined by a spacer that during linearization is removed by either a type IIS or type IIB RE. During this spacer-removal linearization (SRL) these endonucleases remove their own recognition sequences from the vector leaving no undesired nucleotides in the final construct. SRL is combined with In-Fusion™, which is simple and efficient.

## Results

### Creating plant transformation vectors

**Figure 1:**
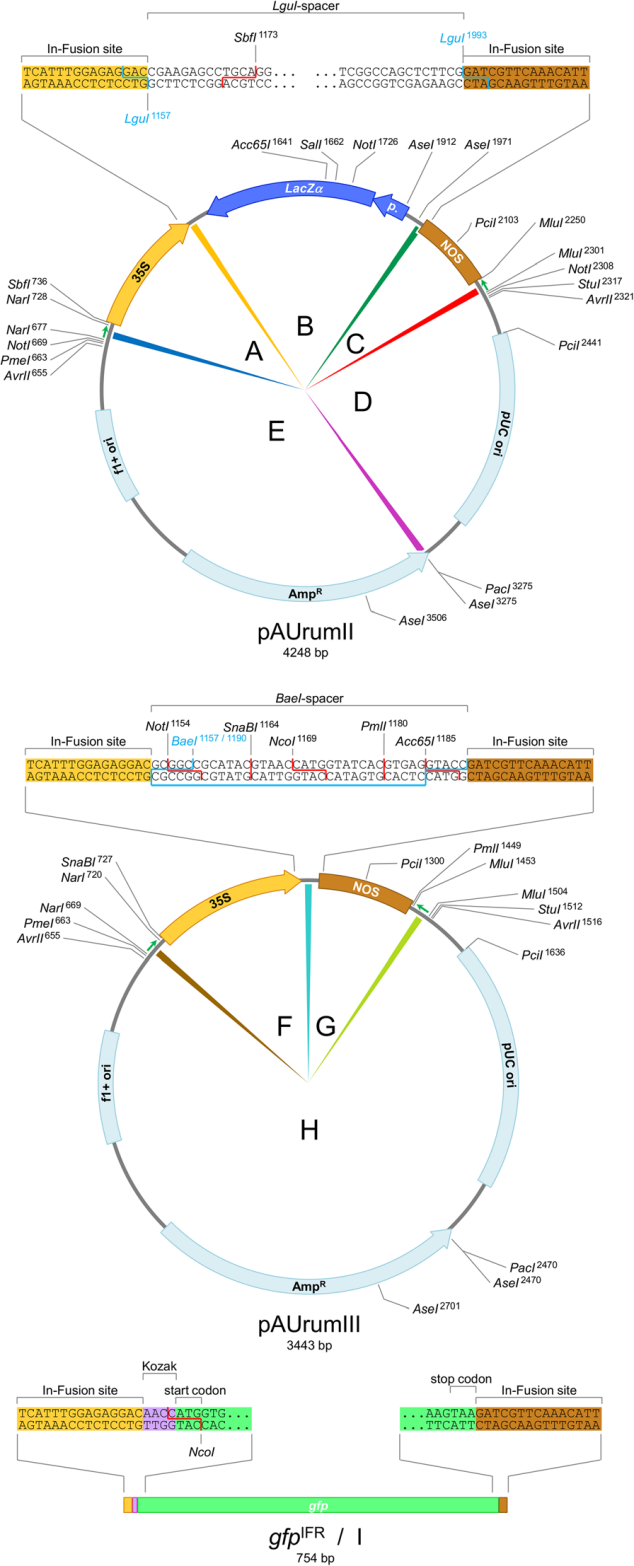
**Maps of vectors and insert.** pAUrumII prepared from the fragments A–E, pAUrumIII from F–H and the In-Fusion™-ready gene for green fluorescent protein, *gfp*^IFR^ (the I fragment) are mapped with selected RE cleaving sites and nucleotide sequences. Colors of the fragment separators of the two plasmid maps correspond to the indications of sequence homology in Figure 3. Small green arrows indicate randomly generated sequences for optimized primer annealing (not used here) and the sequences can be removed with *NarI* and *MluI*.

As the first two of a collection of plant transformation vectors prepared for SRL used with In-Fusion™ cloning, pAUrumII and pAUrumIII were designed (Figure 1). They were prepared for gold particle bombardment and have thus no active elements in connection with plant cell entry like e.g. border sequences of binary vectors for *Agrobacterium tumefaciens*-mediated transformation. The 35S promoter of Cauliflower Mosaic Virus was chosen as it is constitutively active throughout most plant tissues. For transcription termination, the nopaline-synthase (NOS) terminator of *A. tumefaciens* was selected.

**Figure 2:**
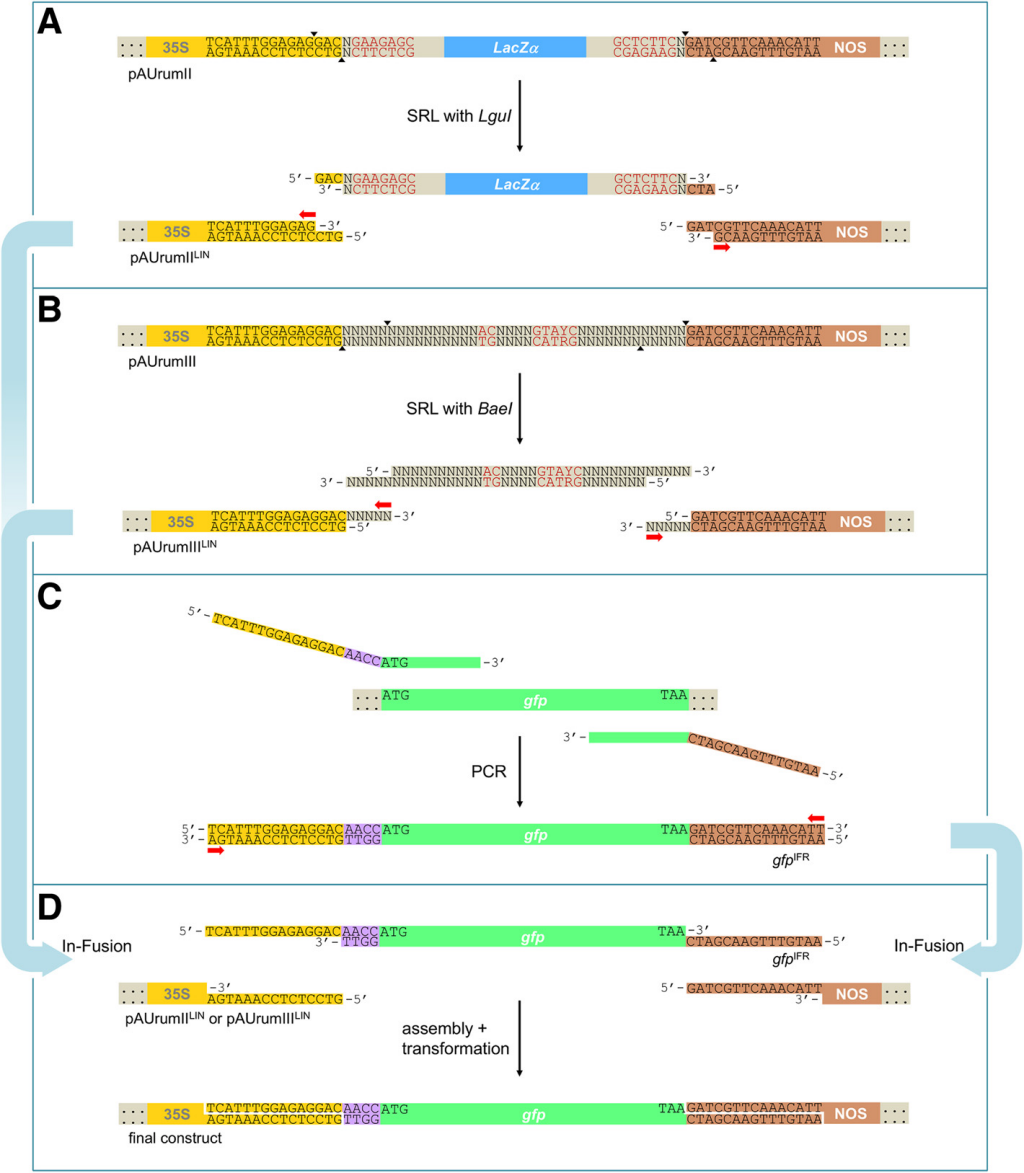
**Overview of SRL used with In-Fusion™ cloning.** Red letters indicate recognition sites and black triangles cleaving sites of *LguI* and *BaeI*. Red arrows indicate where In-Fusion™ enzyme exonuclease activity will occur. **A**. Spacer-removal linearization (SRL) of pAUrumII (to pAUrumII^LIN^) with type IIS RE *LguI*. The spacer holds the *LacZα* coding sequence with its promoter. **B**. SRL of pAUrumIII (to pAUrumIII^LIN^) with type IIB RE *BaeI*. **C**. Extended primers used in PCR amplification of *gfp*. The In-Fusion™-ready product, *gfp*^IFR^, has 15 nt of homology with the 35S promoter 3’-end and 15 nt of homology with the NOS terminator 5’-end. Moreover, it has the monocot Kozak consensus sequence AACC in front of the start codon. **D**. In-Fusion™ reaction and assembly of pAUrumII^LIN^ or pAUrumIII^LIN^ with *gfp*^IFR^ and subsequent transformation of non-ligated construct to *E. coli*, where repair and ligation will occur. There are no unwanted nucleotides in the final construct.

Between the promoter and the terminator, pAUrumII has a spacer of 833 bp containing a recognition site for the type IIS RE *LguI* (5’–GCTCTTCN^∨^NNN_∧_–3’) at both ends with the cleaving sites being the last 3 bp of 35S and first 3 bp of NOS, respectively. So when pAUrumII is linearized with this enzyme, it creates 3 nt 5’-overhangs at these location, while it removes its own recognition sites from the vector (Figure 2A). The nucleotides of these 5’-overhangs are included in the 15 nt overhangs created during In-Fusion™ enzyme treatment. We placed the *LacZα* coding sequence with promoter between the *LguI* sites for detecting carry-over of non-linearized vector, when cloning genes-of-interest.

Five PCR fragments (see Methods and Figure 1), approx. 60 fmol of each, were In-Fusion™ cloned to form pAUrumII. Most of the appearing colonies were blue due to the presence of the *LacZα* coding sequence and 6 of 20 analyzed blue colonies contained a correctly assembled construct, when analyzed with *AseI*, *NotI* and *SalI* (not shown); we did not look into what the remaining 14 colonies contained.

The spacer used in pAUrumIII is much shorter since cleavage at both the promoter and the terminator locations are performed physically by the same type IIB RE, *BaeI*. This enzyme is associated with the sequence 5’–_∧_NNNNN^∨^N_10_ACN_4_GTAYCN_7∧_NNNNN^∨^–3’ (Y = C or T) with the recognition site being located between two cleaving sites. Therefore, when linearizing with *BaeI*, the enzyme creates 5 nt 3’-overhangs both at the 35S 3’-end and the NOS 5’-end, while it removes its own recognition sequence from the construct (Figure 2B). The nucleotides of these 3’-overhangs will be chewed back by the In-Fusion™ enzyme, which then continues chewing back, thus creating the 15 nt 5’-overhangs for assembly. Obviously, the size of a type IIB spacer does not allow the presence of the *LacZα* coding sequence. Among the many optional nucleotides between the two cleaving sites of the spacer, however, we placed a recognition site for *SnaBI* for future exchange of promoter (a similar site is present upstream of the 35S) and a site for *PmlI* for the terminator to be exchanged (downstream of the NOS a similar site is present). Conventional cloning using *NotI* + *Acc65I* can also be performed for direction controlled but not seamless insertion.

Approx. 60 fmol of each of three fragments (see Methods and Figure 1) were In-Fusion™ cloned to form pAUrumIII and since they were all PCR amplified from pAUrumII, which holds the *LacZα* coding sequence in its spacer, any template carry-over would lead to colonies being blue. Few blue colonies were formed and among the many white colonies we found 16 out of 16 analyzed with *AvrII* and *PciI* (not shown) to contain the correct construct.

### SRL and In-Fusion™ cloning of *gfp*

**Figure 3:**
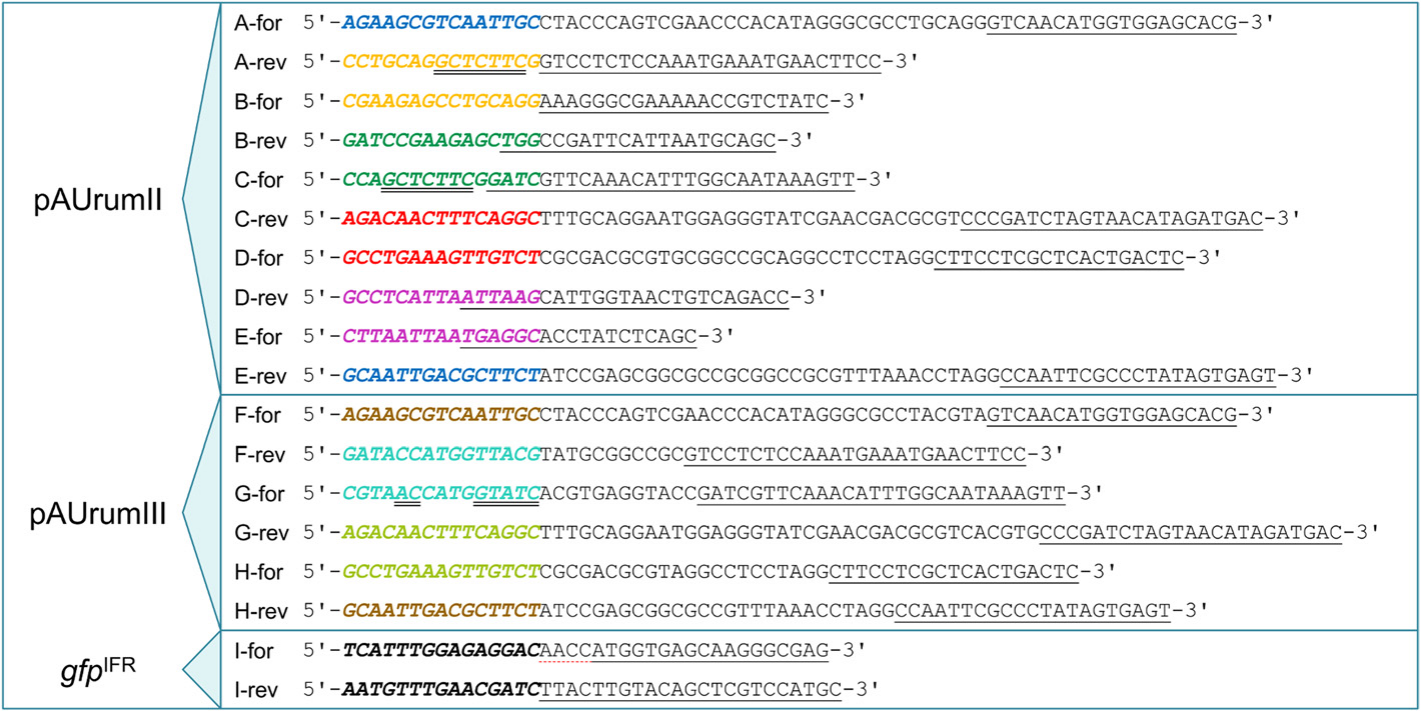
**PCR primer sequences.** The fragment to be PCR amplified (A–I) and the orientation, forward (for) or reverse (rev), are indicated in the primer name in front of the primer sequence. Sequence with a single, black underline represents the part of the primer that anneals to the template. Sequence in bold, italic represents the 15 nt for In-Fusion™ cloning and pairwise coloring (A–H primers) represents homology; the same colors are used in Figure 1, indicating that the fragments are assembled from these homologous sequences, thus forming the two plasmids. Sequence with double underline represents recognition sites for *LguI* (A-rev, C-for) or *BaeI* (G-for), whereas sequence with dashed, red underline represents the monocotyledon Kozak consensus sequence (I-for).

Primers were designed to amplify *gfp*(S65T) [16] and incorporate the monocotyledon Kozak consensus sequence, AACC [17], as well as the 15 bp extensions necessary to facilitate In-Fusion cloning (Figures 2C and 3).

**Figure 4:**
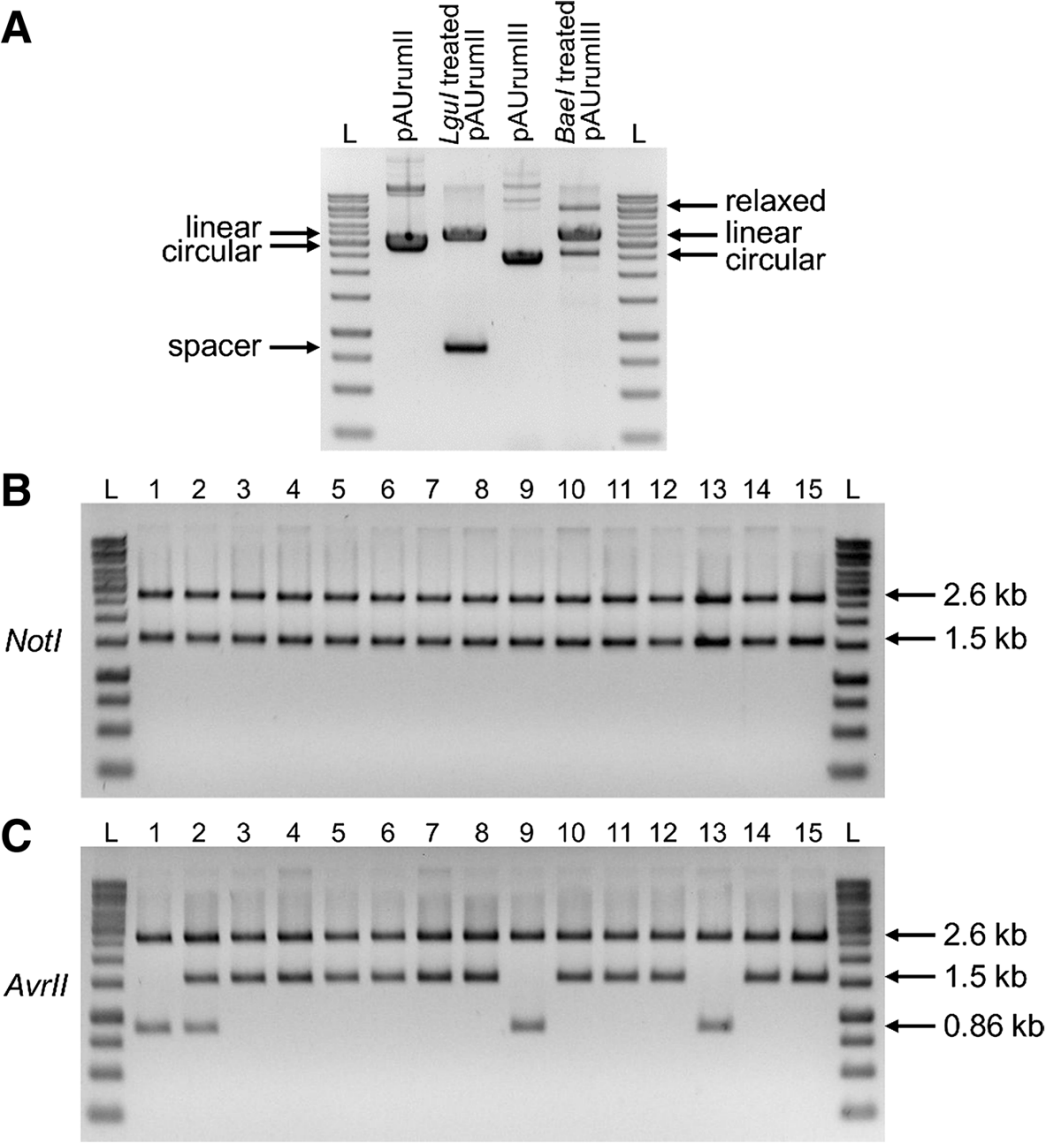
**Vector linearization and RE analysis of cloned *gfp*. A**. pAUrumII in its circular (supercoiled) and *LguI* treated, linear state with the *LguI*-spacer removed next to pAUrumIII in its circular (supercoiled) and *BaeI* treated, linear state. The *BaeI* treated pAUrumIII also appears in a relaxed, circular state. The short *BaeI*-spacer is not visible. **B**. Restriction analysis for pAUrumII-*gfp* with *NotI* on plasmid DNA from 15 colonies. The correct construct is separated in two fragments of 2.6 kb and 1.5 kb, respectively. **C**. Restriction analysis for pAUrumIII-*gfp* with *AvrII*. The correct construct is separated in two fragments of 2.6 kb and 1.5 kb, respectively. The construct separated in a 2.6 kb and a 0.86 kb fragment is pAUrumIII. For all gels, L indicates the O’GeneRuler 1 kb DNA Ladder (Thermo Scientific).

The vectors pAUrumII and pAUrumIII were linearized overnight. From the agarose gel we found no indications of an incomplete *LguI*-digestion of pAUrumII, whereas a small amount of non-linearized (supercoiled) pAUrumIII was left by *BaeI* (Figure 4A). Also an amount of some relaxed, probably nicked, state of circular pAUrumIII was observed.

Insert and linearized vector were equimolarly mixed for In-Fusion™ reaction, and the mixture of non-ligated, assembled fragments was transformed into *E. coli* (Figure 2D). Among colonies from the pAUrumII-*gfp* transformation (n = 299, distributed on three plates) no blue were found. Plasmid mini-preparations of liquid overnight cultures of 15 randomly selected colonies from one plate were analyzed with *NotI* (Figure 4B) and *AseI* + *NcoI* (not shown), and all digestions gave the expected fragments. Blue/white screening was not an option, when selecting colonies from the pAUrumIII-*gfp* transformation (n = 367, distributed on three plates). 15 randomly selected colonies from one plate were analyzed with *AvrII* (Figure 4C) and *AseI* + *NcoI* (not shown), and 11 gave the expected fragments. In four colonies the analysis revealed the presence of pAUrumIII; in one case we found indications that both pAUrumIII and pAUrumIII-*gfp* were present, suggesting that the colony was developed from two single cells.

### Transient *gfp* expression

**Figure 5:**
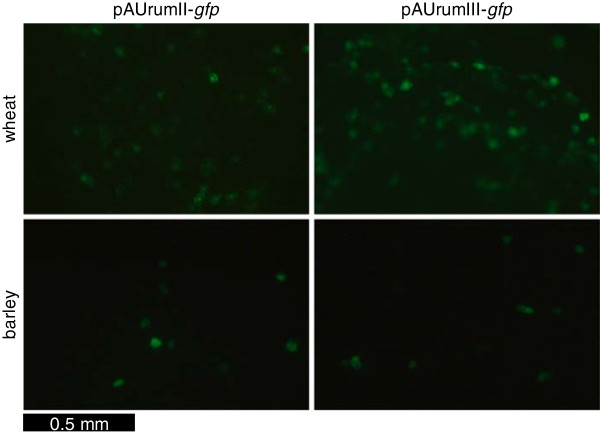
**Transient *gfp *expression in endosperms.** Wheat (upper) and barley (lower) immature endosperms two days after bombardment with pAUrumII-*gfp* (left) or pAUrumIII-*gfp* (right) evaluated under UV light. The bar length represents 0.5 mm. Handling parameters such as positioning of the endosperms in the gene gun influence the outcome of the experiment. Endosperms with *gfp* expression were therefore preferentially selected for the illustration.

The collection of plant transformation vectors to be built is intended mainly for crops like barley and wheat. For this reason pAUrumII-*gfp* and pAUrumIII-*gfp* were tested *in vitro* on wheat and barley endosperm cells. Two days after bombardment, the material was evaluated under UV light in a stereo microscope and it was observed that both constructs worked in both species; the wheat endosperms, however, contained more green glowing cells than those of barley, and for both species not all endosperms contained *gfp*-expressing cells due to uneven quality of the endosperms and/or varying positioning of these in relation to the bombardment (Figure 5).

## Discussion

Today’s cloning of vectors is to a large extent based on the creation of long matching overhangs between the fragments to be assembled. We investigated here the possibility of starting a collection of biolistic plant transformation vectors based on SLIC (specifically In-Fusion™) ready to be cloned with different genes-of-interest, while having the full control of any single nucleotide present in the final construct. For this we used a spacer occupying the cloning site. This spacer is removed during the linearization process by a type IIS or IIB RE, and along with the spacer, the enzymes remove their own recognition sequence as well. Our demonstration that 35S–(Kozak)*gfp*–NOS was efficiently cloned and fully operational in a transient expression study on cereal endosperms, indicates that there is a basis for starting such a collection.

A method that leaves out undesired, short sequences in final transformation vectors may be relevant in the creation of genetically modified crops. Recombining DNA in a way that reduces the presence of redundant sequences should in our opinion be strived for. Especially for intragenesis [10,11], SRL combined with In-Fusion™ cloning has relevance. A range of genes-of-interest could be inserted in a spacer-removal-linearized vector with an appropriate promoter and terminator, all sequences being available within crossable relatives to the species to be transformed. When it comes to cisgenesis [10,11], however, the gene-of-interest must be flanked by its own natural promoter and terminator, and it is, therefore, not possible to prepare promoter–spacer–terminator vectors suitable for different genes. By means of the flanking sites for RE *SbfI*, the 35S promoter can easily be exchanged with a range of different promoters, e.g. a native gene promoter. Such promoter can easily be inserted through In-Fusion™ cloning, while ensuring that the *LguI* and *SbfI* sites are restored from extended PCR primers prior to this step. If, however, one or more sites for *LguI* are present in the new promoter, the spacer needs to be recreated for the use of another type IIS RE (it is irrelevant that a chosen type IIS RE may also cleave within the spacer sequence).

Type IIS REs have successfully been used in connection with seamless genetic recombination (reviewed in [12]). In a sophisticated variant of classical cleaving-ligating cloning, the type IIS RE *BsaI* has been employed [18]. Subcloning a fragment from one vector, leaving recognition sites for *BsaI* in the backbone, into another vector, linearized with the same type IIS RE by removing a fragment holding the recognition sites, has several advantages. Besides having no *BsaI* recognition sites in the final construct, the advantages include the full control of insert orientation, as well as simultaneous ligation in the digestion solution. The method, referred to as Golden Gate, has been further developed to shuffle several DNA fragments in single reactions [19]. Also the type IIB RE *BaeI* has previously been used in an advanced technique for shuffling DNA named sequence-independent site-directed chimeragenesis (SISDC) [20]. In addition to these ligase-dependent methodologies, we demonstrate here the type IIS and IIB REs as a useful means for SRL used with In-Fusion™ cloning, which has the advantage of being ligase-independent and thus relatively rapid. The applications of this method are in our opinion not limited to plant science, but can also be relevant in connection with expression vectors for *E. coli* and yeast. The principle was shown useful in connection with protein expression in *E. coli*[15]*.*

Omitting undesired nucleotides in In-Fusion™-cloned final constructs could be performed in different ways [12]. In the In-Fusion™ cloning, the insert could be received by a vector, which is not linearized from a circular state, but instead prepared as a linear PCR product from a circular or linear template ( [21], and as proposed by the In-Fusion™ manufacturer). The primers used to generate this linear vector will determine, which nucleotides will be flanking the insert, and a construct with similar seamlessly joined promoter, gene-of-interest (insert) and terminator, as we demonstrate by using SRL with In-Fusion™ cloning, can be achieved. PCR amplifying the insert-receiving vector, however, may lead to PCR mistakes in the vector backbone, and this risk is increased with the vector size. The linearization option may lead to contamination with carry-over of non-linearized vector, when transforming *E. coli* with the In-Fusion™-cloning mixture; creating a linear vector from PCR amplification, however, may similarly lead to contamination with carry-over of template-vector. Template-vector may be degraded prior to transformation with a type IIM RE like *DpnI*, which only digests methylated DNA and thus spares PCR products, but addition of this step to the procedure contributes to the over-all working time. So, from this point of view it is not clear, which is preferable; linearization or PCR amplification of the vector. Another way of cloning a gene-of-interest seamlessly between a promoter and a terminator, possibly with few extra desired nucleotides flanking it, is to insert promoter and terminator as separate fragments simultaneously with the gene-of-interest, *i.e.* a 4-fragment In-Fusion™ cloning. SLIC is, due to the long, unique overhangs, suitable for cloning more than one fragment; the efficiency of the cloning, however, most likely decreases with every fragment added to the cloning, and the risk of contamination with carried-over template increases. Besides, the risk of introducing PCR errors in the promoter and terminator is also present.

SRL takes advantage of the special characteristics of type IIS and IIB REs, having their recognition site separated from their cleaving site/sites. *LguI* and *BaeI* were chosen among several other type IIS and IIB REs and the main criteria was that recognition sites for the enzyme in question were not present outside the spacer sequence. It seems that *BaeI* does not digest the vector to the same level as *LguI*, for which no visible non-linearized vector was present in the agarose gel (Figure 4A). The possibility that this holds true for other type IIB REs, as a possible consequence of the cleaving process being more complicated than for type IIS REs, or if the choice of *BaeI* was just unfortunate, was not examined.

The use of two recognition sites for a type IIS RE rather than one for a type IIB gives a size-independent spacer. We took advantage of this and placed the coding sequence of *LacZα* with its promoter in the *LguI*-spacer. In this way it is possible to detect colonies transformed with non-linearized vector, which may be present even after an overnight digestion. The type IIB spacer does not allow such a screening system. For both pAUrumII and pAUrumIII, the linearized and the circular (supercoiled) vector run close to each other on an agarose gel (Figure 4A), and because *BaeI* and possibly other type IIB REs do not digest completely, it would especially here be an advantage to have the possibility of blue/white screening.

SLIC and the similar methods that rely on the creation of long matching overhangs allow the use of unpurified PCR products in the assembly process. But the relatively easy step of purifying linearized vector and PCR products is probably worth doing in order to reduce the level of false positives. The time saved by skipping these steps is easily used on doing more plasmid mini-preparations and RE analysis to find the right clone.

## Conclusions

All nucleotides flanking an insert in a plant transformation vector can be controlled from the combination of spacer-removal linearization (SRL) of the insert-receiving vector and sequence and ligation-independent cloning. We demonstrate that both type IIS and IIB restriction endonucleases can be used to remove the spacer occupying the cloning site of the vector; it seems, however, that the use of a type IIS has more advantages for an efficient cloning outcome. Based on our findings, we believe that the SRL system can be useful for the generation of expression vectors such as plant transformation vectors.

## Methods

### PCR

All PCRs were carried out with Herculase II Fusion DNA polymerase (Agilent) or Velocity DNA polymerase (Bioline) in agreement with general protocol guidelines. PCR primers were purchased from Invitrogen, Germany. As many of the primers had a relatively long sequence attached to the template-matching sequence (Figure 3), 6–8 initial cycles with 58°C annealing temperature were performed followed by an increase in temperature to 63°C for an additional 30 cycles.

### Purification

To avoid carry-over of template DNA in the cloning reactions, all PCR fragments were purified from a 1.2–1.5% (w/v) agarose gel with ethidium bromide or GelStar (Lonza) staining and subsequent spin column recovery (NucleoSpin Gel and PCR Clean-up, Macherey Nagel). Likewise, linearized vectors were purified from agarose gel followed by spin column recovery to eliminate the presence of non-linearized vectors in the *E. coli* transformation solution.

### Creating plant transformation vectors

The plant transformation vector pAUrumII was created from five PCR produced fragments (Figures 1 and 3). Fragment A contained the Cauliflower Mosaic Virus (CaMV) 35S promoter and fragment C contained the nopaline synthase terminator (NOS) of *Agrobacterium tumefaciens*. Promoter and terminator were linked by fragment B holding the *LacZα* coding sequence with its promoter. Fragment B was flanked by recognition sequences for the type IIS RE *LguI* with cleaving sites pointing outward. Fragments B, D and E were amplified from a pBluescriptII SK + (Agilent) template, and D combined with E forms the vector backbone. We wanted to introduce a recognition site for the RE *PacI* in the beta-lactamase (ampicillin resistance) gene for having the possibility of linearizing the final vector opposite the expression cassette if desired (not relevant here); the *PacI* site, however, does not disturb the ORF of the beta-lactamase gene. In the 15 common base pairs (for In-Fusion™ cloning) of fragments D and E the *PacI* site was introduced. Fragment D, closest to the terminator fragment C, was amplified so that the existing recognition site for *LguI* of pBluescriptII SK + was not included. The 35S–*LguI*-spacer–NOS cassette was flanked by two short, randomly generated sequences (R package version 2.14.0) with optimized PCR-primer sites prepared for tracking full-lengths inserted expression cassettes in the genome of regenerated plants; hence the great length of some of the PCR primers for this construct. In the present study, however, these sequences were not utilized, as we tested the construct only transiently.

All five fragments, ranging in size from 314 to 1,683 bp, were joined in equimolar amounts (approx. 60 fmol of each) in 10 μL 1X In-Fusion™ enzyme mix (Clontech) for 15 minutes reaction at 50°C before assembly on ice. 50 μL competent Stellar (Clontech) *E. coli* cell solution in 2.2 mL microcentrifuge tubes on ice were transformed with 2.5 μL of the In-Fusion™ reaction solution according to the manufacturer’s recommendations before spreading on LB agar plates (100 mg/L ampicillin, 100 mg/L X-gal) for overnight incubation at 37°C.

Plasmid mini-preparations (FastPlasmid Mini Kit, 5Prime) of overnight LB cultures (100 mg/L ampicillin) of randomly selected blue colonies were analyzed with the REs *AseI* (New England Biolabs), *NotI* and *SalI* (both Fermentas).

Three fragments, F, G and H, for the transformation vector pAUrumIII were PCR amplified using pAUrumII as template (Figures 1 and 3). F holds the 35S promoter and G the NOS terminator. The relatively short type IIB RE spacer, with a recognition site for *BaeI*, was introduced between F and G from the PCR primers. Fragment H corresponds to D + E and when combined with the promoter and terminator fragments, only few nucleotides make pAUrumIII differ from pAUrumII outside the 35S–*BaeI*-spacer–NOS cassette; these are RE recognition sites irrelevant for construct functionality.

The In-Fusion™ cloning was carried out as for pAUrumII and plasmid mini-preparations of overnight cultures of randomly selected white colonies were analyzed with *AvrII* and *PciI* (both New England Biolabs).

A plasmid midi-preparation (NucleoBond Xtra Midi/Maxi, Macherey Nagel) of a selected positive clone of each of pAUrumII and pAUrumIII were verified by sequencing at Eurofins MWG Operon, Germany.

### Linearizing plant transformation vectors

Five micrograms of pAUrumII was digested 15 h at 37°C using 6 U/μg of the type IIS RE *LguI* (*SapI*) (Fermentas) in a 100 μL reaction solution. In another 100 μL reaction solution containing 20 μM S-adenosyl methionine, 5 μg pAUrumIII was digested 15 h at 25°C using 10 U/μg of the type IIB RE *BaeI* (New England Biolabs). Both digestions were terminated at 65°C for 20 min, and to further reduce the risk of transforming with empty vectors, both the linearized vectors, pAUrumII^LIN^ or pAUrumIII^LIN^, were treated with 0.6 U/μg calf intestinal alkaline phosphatase (Invitrogen) for 30 min at 37°C before purification.

### Preparing *gfp* for insertion

To evaluate the constructs, the gene for a green fluorescent protein, *gfp*, was chosen for insertion (we use the gene for sGFP(S65T) [16], originating from plant transformation vector pVec8-GFP [GenBank: FJ949107.1]). Using the I-primers (Figure 3), we prepared an In-Fusion™-ready fragment by PCR, *gfp*^IFR^, *i.e.* the ORF of *sgfp*(S65T), with the monocotyledon Kozak consensus sequence AACC in front of the ATG start codon, extended at both ends with nucleotide sequences corresponding to the last 15 nucleotides of the 35S promoter, TCATTTGGAGAGGAC, and the first 15 of the NOS terminator, GATCGTTCAAACATT, respectively (Figures 1 and 2C).

### In-Fusion™ cloning of *gfp*

Approx. 60 fmol of *gfp*^IFR^ were joined in equimolar amounts with pAUrumII^LIN^ or pAUrumIII^LIN^ in 10 μL 1X In-Fusion™ enzyme mix, and reaction and transformation occurred as described for the vectors above. After the 1 h shaking of the transformed bacteria, a 1:100 dilution was prepared in S.O.C. medium and 100 μL were spread on each of three LB agar plates (100 mg/L ampicillin, 100 mg/L X-gal) before incubation overnight at 37°C.

Plasmid mini-preparations of overnight LB cultures (100 mg/L ampicillin) of randomly selected single white colonies were analyzed with REs *NotI* for pAUrumII-*gfp*, *AvrII* for pAUrumIII-*gfp* and *AseI* + *NcoI* for both constructs (all New England Biolabs).

A plasmid midi-preparation of a selected positive clone of each of pAUrumII-*gfp* and pAUrumIII-*gfp* was made and correct gene insertion was verified by sequencing at Eurofins MWG Operon, Germany.

### Preparation of plant material

Immature seeds of barley, *Hordeum vulgare* cv. ‘Gunhild’ (NordGen) and wheat, *Triticum aestivum* cv. ‘AvocetYr10’ from green house were collected and sterilized 10 min in a sodium hypochlorite solution of about 1.5% active chlorine (VWR) and 0.05% (v/v) Tween 20 (Sigma) before three times wash in sterile Milli-Q water. Sterilely handled, the seeds were cut open with scissors at the embryo end and endosperms were squeezed out and placed 3 x 5 in the center of Petri dishes containing 1X Murashige & Skoog medium including modified vitamins (Duchefa Biochemie, pH = 5.8) supplied with 36.5 g/L mannitol, 20 g/L maltose, 3.5 g/L phytagel, 1.5 g/L ammonium nitrate, 1 g/L casein hydrolysate, 690 mg/L L-proline and 150 mg/L myo-inositol. One plate each of barley and wheat endosperms was prepared for each of the two *gfp*-constructs. The endosperms were stored for about an hour in darkness until bombardment.

### Gold particle bombardment for transient *gfp* expression

The gold preparation and coating procedure below was based on available procedures [22]– [24]. Twenty milligrams 0.6 μm gold particles (Bio-Rad) were heated overnight at 180°C. After cooling, the particles were suspended in 1 mL 2-propanol and transferred to a microcentrifuge tube, ultra-sonicated for 2 min and incubated at room temperature for 15 min before another minute of ultra-sonication followed by 1 min of centrifugation at 13,000 g. The supernatant was discarded and the particles were washed three times in 500 μL sterile Milli-Q water with ultra-sonication for 2 min and centrifugation at 13,000 g for 1 min each time. Gold particles were resuspended in 670 μL sterile Milli-Q water, vortexed for 2 min and stored at -20°C in 50 μL aliquots in microcentrifuge tubes.

For each of the two plasmids, a 50 μL gold particle aliquot was thawed and ultra-sonicated for 2 min. With the tube lid open, the particles were vortexed gently, while 5 μg plasmid in aqueous solution and subsequently 50 μL 2.5 M calcium chloride premixed with 20 μL 0.1 M spermidine were added. The vortexing was continued 1 min followed by centrifugation at 13,000 g for 5 sec. The DNA coated particles were washed in 140 μL 2-propanol and spun again. Finally, they were suspended in 50 μL 2-propanol and kept few minutes on ice until bombardment.

Bombardment was performed at 25 inHg vacuum and 1,100 psi helium pressure using a Bio-Rad PDS-1000/He Biolistic gene gun in a sterile bench. The DNA-gold mixture was ultra-sonicated for 3 x 1 sec and 10 μL droplets were left on macrocarriers, which were then kept in closed Petri dishes on a non-vibrating surface, *i.e.* outside the sterile bench, for slow 2-propanol evaporation to occur, ensuring an equal, smooth distribution of gold particles. The bombarded endosperms were kept in darkness in a 23°C incubator for two days before *gfp* expression was evaluated under UV light in a stereo microscope (Leica WILD MZ8).

## Competing interests

The authors declare that they have no competing interests.

## Authors’ contributions

RK developed SRL, conducted the experimental part and wrote the manuscript. CRI contributed to the development of the gold coating procedure, for which she also found the relevant literature. CKM suggested the use of the *LacZα* coding sequence in the *LguI*-spacer. All authors agreed on the final appearance of the manuscript after careful review.
